# Medicinal and commercial uses of ostrich products in Tanzania

**DOI:** 10.1186/s13002-017-0176-5

**Published:** 2017-08-23

**Authors:** Flora Magige, Eivin Røskaft

**Affiliations:** 1 Department of Zoology and Wildlife Conservation, University of Dar es Salaam, P.O. Box 35064, Dar es Salaam, Tanzania; 2 Department of Biology, Norwegian University of Science and Technology, Realfagbygget, -7491 Trondheim, NO Norway

**Keywords:** Ostrich, Livelihood, Serengeti, Illegal hunting, Game ranching

## Abstract

**Background:**

Traditional communities have been utilizing animal products for numerous purposes and have for a long time contributed to the accumulation of world knowledge. Local people in Tanzania and elsewhere in Africa, have been using birds including ostriches as pets or their products such as meat, eggs as food; their body parts such as feathers, bones and hide for ornaments but more importantly have used such products in traditional medicine and rituals. Nevertheless, there is a general lack of information about the differences that exist between local people with different cultures, and the best use of such products to improve their livelihoods. This study aimed to determine the use of ostrich products among people residing around Serengeti National Park and explore the potential of improving livelihoods through game ranching.

**Methods:**

Use of the products was compared between that of agriculturalists with long hunting traditions in the Serengeti District to the west of Serengeti National Park (SNP) and the largely pastoral community in the Ngorongoro District to the east by using semistructured questionnaires in June 2006.

**Results:**

A total of 115 respondents were interviewed, and the majority (74.5%) in the Serengeti district admitted that ostriches were mainly hunted for their products by snares, while in the Ngorongoro district, 98.2% of the respondents said that villagers only gathered products such as feathers and eggs. Ostriches were hunted for food, ornamentation, medical and economic purposes, and eggs and oil, which are believed to have medicinal properties, were used for the treatment of various ailments, including asthma. This indigenous knowledge of the medicinal value of ostrich products must be integrated with scientific knowledge to prove the supposed medical efficacy of the products. Ostrich products also had market value and were thus sold to the villagers.

**Conclusion:**

Since it has been found that ostrich products are commercially used, legal establishment of markets through game ranching, might improve local livelihood while simultaneously promoting the conservation of ostriches, whose populations are declining, by reducing hunting pressure. Ostrich farming and conservation education programs are recommended.

## Introduction

Inhabitants of traditional communities, including gatherers, livestock keepers or agriculturalists devoted their time to understanding their environment on which they earned their livelihood. Indigenous local people actually have good knowledge about their biological environment and they have been passionate to it. Agriculturists residing to the west of Serengeti National park (SNP) and the pastoralists Maasai to the east are an interesting group of people to study in terms of cultural biological knowledge. The protected area they are bordering to was established more than 5 decades ago to protect it from the growing human population influence. Growing human populations have been accompanied with increased human activities that threatens bird populations. Such threats include habitat fragmentation, habitat loss and degradation [1], road kills [2] and illegal hunting [3]. Illegal hunting of birds has been reported to affect their populations with immediate ecological consequences [4]. Illegal hunting is common in Africa and mainly practiced as a means to obtain protein [3, 5, 6] and to fulfil cultural and social needs [3]. Illegal hunters are typically poor local men in their middle ages [7], and they commonly use different types of traps [8]. Although some bushmeat and other animal products are consumed by the hunters, most are sold in their respective villages [9]. Recently, the trade has increased due to the increasing human population, and wildlife harvesting has, in many cases, become unsustainable [10, 11]. Some animal products are sold to middlemen, who transport them to more distant urban centres or even across borders to nearby countries through black markets (personal observation). Illegal hunting has resulted in a major decline in wildlife populations in various parts of Africa [5, 12, 13], and if it continues unabated, it is likely to lead to the extinction of many animal species [14].

Tanzania is among the countries most highly affected by illegal hunting in protected areas making it an important country for investments in conservation [6]. Illegal hunting of different taxa of animals including birds is being practised for food, income, ornamentation and medicinal purposes in rural communities. However, little research has explored the possibilities of improving the livelihoods of the communities through alternative uses of such products. This study focuses on the utilization of products from the world’s largest bird, the ostrich (*Struthio camelus*). This animal is an important game bird, not only in Tanzania and Africa, but also in other countries in the world as a domesticated species. Although the distribution of the ostrich has severely declined in most African countries over the past decades [15], it is categorized as a species of ‘Least Concern’ in the International Union for Conservation of Nature (IUCN) Red List [16]. Ostrich declines could be attributed to mechanized agriculture, overgrazing, illegal hunting and drought ([15]. Over 90% of the current ostrich population in Africa are kept on farms or in zoological gardens while the remainder live in the wild [15].

Ostrich products such as meat and eggs have been used for food; feathers for ornaments; oil for medicinal purposes [10, 17–19] and even traded since antiquity [20]. So ostriches do provide an important ecosystem service. Ostrich skins are an especially valuable commodity [21, 22]. In Tanzania, ostrich products are mostly utilized locally (personal observation), but in the US, South Africa, Brazil, Germany and New Zealand, there are large ostrich ranches and products from ostriches are in high demand by the food, clothing and cosmetics industries [21].

This study aimed to investigate the use of ostrich products in villages bordering the Serengeti National Park (SNP) and explore the possibility of establishing ostrich farms. Such farming has the potential to improve the livelihoods of local people while simultaneously reducing hunting pressure in this protected area. We tested the hypothesis that ostrich products are commonly used by the local communities surrounding SNP, but we predicted differences among different ethnic groups in the use of products such as eggs, feathers, meat and oil.

## Methods

### Study area

**Fig. 1:**
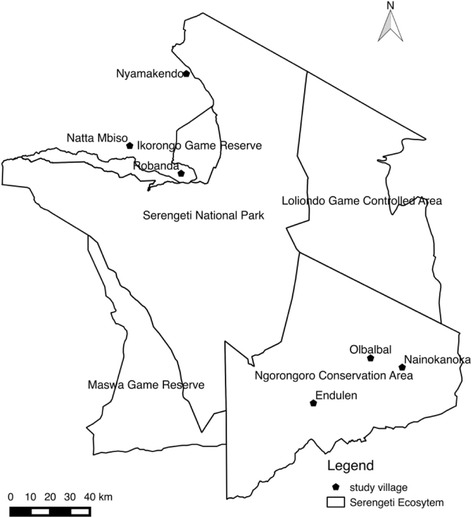
Map of the Serengeti ecosystem showing study villages; thick lines denote the protected areas

The study was conducted in villages surrounding SNP (14,763 km^2^). The villages included Robanda, Natta Mbiso and Nyamakendo on the western side of SNP, where the respondents came from the Ikoma, Natta and Kurya tribes, respectively. On the eastern side, the villages included Olbalbal, Endulen and Nainokanoka (all located in the Ngorongoro Conservation Area (NCA) (8300 km^2^), which lies east of SNP and is contiguous with the southern Serengeti plains) (Fig. 1). Respondents from Ngorongoro district were all Maasai by tribe. The people in the Serengeti district practice hunting, cultivation and livestock husbandry, while the people in the Ngorongoro district largely rely on livestock keeping.

Data on the utilization of ostrich products were collected through questionnaires in the above mentioned villages in June 2006, and a total of 115 respondents from approximately 5% of the total households in the selected villages were interviewed. Random sampling was used in selecting the first household whereby only one member of the family was interviewed. Thereafter, snow ball technique [23] was used to select the subsequent interviewees after the first interviews. The snow ball technique involves using research participants in recruiting other participants for a research or study. Attempts were made to establish a friendly relation with the local authority and the villagers so that they would participate in the research since they were aware that utilizing wildlife products without permission is illegal. In addition, appointed local people accompanied us in administering the questionnaires because their presence reduced the tension and suspicion of the interviewees to the researchers. However, in some cases, the residents refused to provide information or gave incomplete information about the subject researched. Before each interview, we introduced ourselves and the nature and objective of the research were explained, and the interview began after getting their consent. In total 115 respondents were interviewed (52 females and 63 males). The questions were semi structured which allowed free interview and informal conversation [24]. Only one member per household aged 18 years and above was interviewed. Both males and females were interviewed, however in some families when the father is around the mother is not allowed to talk and therefore in some situations the father was interviewed because he was at home. Standardized questionnaires were used and they contained questions on (i) personal information, (ii) economic activities (iii) reasons for hunting, (iv) hunting methods, (v) the number of ostriches hunted per week, (vi) when do the ostriches start breeding, (vii) which season of the year the ostriches were seen in large numbers and (viii) the uses of ostrich products, and (ix) buyers of the products. The ethical approval for the study was obtained from the Ethics Committee of the University of Dar es Salaam.

### Statistical analyses

SPSS 20 and *Excel 2010* were used for the statistical analyses. Chi-square (χ^2^) tests on contingency tables were used to test for an association between culture and wild meat-eating habits, and descriptive statistics were used to determine the percentage frequency of ostrich product utilization. Mann Whitney U tests were used to compare the prices of different ostrich products between the two districts. The results were considered significant at p ≤ 0.05 and means are presented as mean ± SD.

## Results

A total of 115 respondents were interviewed, 56 from the Ngorongoro District and 59 from the Serengeti District. All respondents in the selected villages of the Ngorongoro district were Maasai while in the villages of the Serengeti district respondents were of different tribes (Ikoma (*N* = 22), Natta (*N* = 17), Kurya (*N* = 18) and Sukuma (*N* = 2)). In the Serengeti district, 74.5% of the respondents admitted that ostriches were hunted, while 13.6% said people collected eggs and feathers. In Ngorongoro, 98.2% of the respondents said that villagers did not hunt but rather collected eggs and feathers. In general, 93% of the interviewees responded that ostrich products were used, but 7% said they did not know whether the products were used or not because they did not utilize them themselves. However, even those respondents who said that the products were being used did not admit to being consumers. A wide variety of weapons were used for hunting; snares were the most common in the Serengeti District, while people in the Ngorongoro District relied on the collection of eggs and feathers (Table 1).

**Table 1:** Means used to illegally hunt ostriches in the Serengeti and Ngorongoro Districts (n = sample size), Tanzania, 2006

Means of obtaining ostrich products:	Serengeti (%)	n	Ngorongoro (%)	n
None	11.7	7	1.8	1
Snare	47.5	28	0.0	0
Collection of eggs & feathers	13.6	8	98.2	55
Spear	11.9	7	0.0	0
Rope	8.5	5	0.0	0
Bow & arrow	3.4	2	0.0	0
Gun	3.4	2	0.0	0

**Fig. 2:**
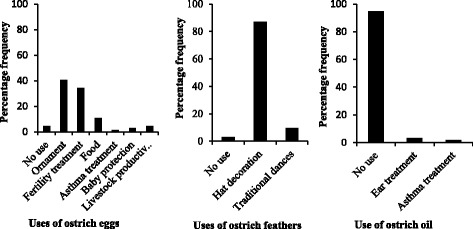
Percentage frequency of the different uses of ostrich products in the Ngorongoro District, Tanzania, 2006

**Fig. 3:**
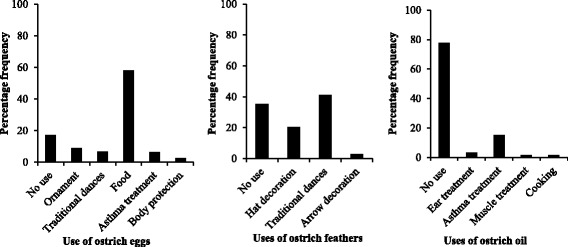
Percentage frequency of the different uses of ostrich products in the Serengeti District, Tanzania, 2006

Some of the people (*N* = 33) from the Serengeti District responded that ostriches were hunted for meat, but the rest said that ostriches were not hunted (*N* = 26). In Ngorongoro, none of the respondents admitted to hunting the bird for meat (*N* = 56), but approximately 91% claimed that eggs were the most preferred product and that they primarily relied on collecting eggs rather than hunting. The test for an association between hunting culture and eating wild meat showed a significant relationship between villages with a hunting culture and wild meat consumption (*X* ^*2*^ = 43.93, df = 1, *p* < 0.001). This indicates that the Serengeti District inhabitants, which have a long culture of hunting and eating wild meat, hunted ostriches for meat. The utilization of ostrich oil was higher in the Serengeti District than in the Ngorongoro District (*X* ^2^ = 8.63, df = 1, *p* = 0.003), and the people in Ngorongoro district used eggs (*X* ^2^ = 6.67, df = 1, *p* = 0.01) and feathers (*X* ^2^ = 22.61, df = 1, *p* < 0.001) at a significantly higher rate than in the Serengeti district. In general, ostrich products such as eggs, feathers and oil were utilized differently in the two districts (Figs. 2 and 3). Ostrich eggs were commonly used as food in the villages of the Serengeti district, but such eggs were mostly used for decoration or as a fertility treatment, and rarely for food, in Ngorongoro district. Eggs are also used to protect children from bad spirits and to increase livestock productivity. The feathers were used for decoration during traditional dances and to decorate the hats of circumcised youths, and oil was used sparingly as an ear and asthma treatment (Fig. 2).

In addition to the use of eggs as food in the Serengeti district villages, the egg shells were hung in houses as ornaments or made into beads for use as decoration during traditional dances. The eggs were also used as treatment for asthma and to protect bodies from bad people, and feathers were used to decorate houses (hung or stuck on the walls) or during traditional dances. Hunting arrows were also decorated with ostrich feathers. Although the use of oil was higher in the Serengeti than in the Ngorongoro District, the product was used infrequently to treat ears that ache or leak pus, muscle spasms and asthma. It was sometimes used for cooking in the Serengeti district. In terms of medication, ostrich products were either taken alone or in combination with medicinal herbs in both districts. Although the respondents in the Serengeti district came from different tribes, there was no difference in the use of ostrich products as the people in those tribes are culturally similar.

With regard to the trade of ostrich products, 44% of all respondents replied that people in their villages had sold ostrich products. Both ostrich eggs and meat were sold in the villages and hotels located within the Serengeti District, but only eggs were traded in the Ngorongoro District. We present the values of ostrich products in US dollars ($), and in the Serengeti district villages, the price for 1 kg of meat ranged from $0.40 – $3.50, mean (SD) = $1.36 ($0.99, *n* = 10). The price for an egg varied from $0.10 to $3.80, mean (SD) = $1.24 ($0.01, *n* = 16), and from $0.50 - $0.80, mean (SD) = $0.56 ($0.10, *n* = 7), in Serengeti and Ngorongoro Districts, respectively. However, there was no significant difference in egg prices between the two districts (MWU = 33, n_1_ = 7, n_2_ = 16, *p* = 0.13). Feathers were not traded in either district, and surprisingly, no one reported the use of ostrich leather, which currently has a high economic price in the world market.

## Discussion

In this study, ostriches were found to be hunted in both districts, but the hunting frequency was higher in the villages on the western side of the national park than in the villages to the east. The people on the western side have a long history and culture of hunting wild animals using a variety of hunting tools. In the Serengeti district, illegal bush-meat hunting is predominantly done with snares and normally targets large herbivores including ostriches [7, 8], but snares are not selective and may trap individuals of the wrong sex or juveniles of the target species [8]. Maasai are not hunters and traditionally do not consume wild meat, but due to the influx of people from neighbouring regions, hunting is currently being practiced and the eating of wild meat has begun at a small scale (personal observation). The hunting culture of the Serengeti people had led to more significant utilization of meat, as well as oil, than that in the Ngorongoro District, where people indicated high utilization of eggs and feathers that were mainly collected in the wild. Wild meat and eggs form an important component of the diet in most areas of Sub-Saharan Africa [5, 25, 26].

As predicted, cultural differences between the two districts led to differences in the use of ostrich products, although there were some overlaps. The people in both areas utilized eggs for food, made beads from egg shells for use as ornaments, mixed egg yolk with body cream to drive away evil spirits, and used feathers and egg shells for decorations during circumcision ceremonies or traditional dances. Another overlap was in the utilization of ostrich products for medicinal purposes based on indigenous knowledge; both groups utilized ostrich eggs and oil to treat asthma and earaches. The difference was that, in Ngorongoro, the people believed that putting egg yolk in feed increased livestock productivity and applying the yolk on the stomachs of women treated infertility, while in the Serengeti district villages, ostrich oil was used to treat muscle spasms. This indigenous knowledge of the medicinal value of ostrich products must be integrated with scientific knowledge to investigate the efficacy of the products. Ostrich oils, for example, have been found to contain varying levels of compounds that may confer therapeutic benefits, including antioxidant properties when ingested [27], and they are widely used in the cosmetics and pharmaceutical industries [28]. It is interesting that in different parts of the world, grease and oil obtained from wild birds is used for massage in muscle aches and in cases of burns [27].

In general, every ostrich product in the study area was put to use, and some were mainly traded among the villagers, supporting the results obtained by other researchers [26, 29, 30]. There is a growing demand for ostrich meat as a healthier alternative to beef. A number of beef producers in Europe, the United States, South Africa, Brazil, Germany, New Zealand and Canada have recently switched to ostrich farming [21, 31]; South Africa dominates the international market for ostrich products, exporting substantial quantities of meat, hides and feathers to the U.S. and other countries [20]. Ostrich meat is characterized by high polyunsaturated fatty acid content, low saturated fatty acid content, and a low level of cholesterol [32]. Therefore, ostrich meat is one of the healthy meats although not all saturated fatty acids elevate the level of cholesterol, Stearic acid, for example, has been shown to lower LDL (low density lipoprotein) cholesterol [33].

Ostrich farming is advantageous over rearing livestock in that less water is required; the growth rate is faster, and the reproductive rate is higher [31]. Cattle cannot tolerate drought, and a good example of that is 2011, when the eastern part of the Serengeti ecosystem was affected by severe drought, leaving many cattle dead at a huge loss to the livestock keepers (personal observation). In Zimbabwe, ostrich farming is seen to be more profitable than raising cattle and an important source of rural employment [20]. In Tanzania, ostrich farming was practiced at Oldonyo Sambu in Arusha, but the farm failed for unknown reasons. In recent decades, there has been a growing interest in ostrich farming that provide healthy meat and valuable skins, feathers, eggs and oil [29, 34–36]. Although, Tanzaniastill boasts a good number of ostriches in the wild and in addition the Serengeti ecosystem still sustains large concentrations of herbivores where illegal killing of ostriches and other large birds is done opportunistically [7], the population of ostriches might be declining due to incidental killing and collection of eggs. The bird is among the Tanzanian government trophy whereby killing or capturing it without a license or permit is strictly prohibited [37]. Therefore, there is a need to conserve and protect the species with the committed involvement of the communities surrounding SNP.

At the onset of incubation, some of the eggs, which are believed to belong to minor females, are ejected from the nest [26, 38, 39], but ostriches also sometimes randomly lay eggs as ‘singletons’ on the ground away from the nests when disturbed [40]. These ejected and singleton eggs are never incubated but left unattended and ultimately decay or are predated. Therefore there is a good chance for communities residing near the protected area to seek permits for collecting such eggs and capitalize on the opportunity to establish ostrich farms and thus reduce pressure on the wild population. However, control by the park authority should be in place so that no just any eggs (nests) are taken. In addition, it is farming, which could help to improve the post hatching survival following the differential predation on males [41] coupled with nest and brood predation [40].. The villages bordering Serengeti National Park (SNP) and Ngorongoro Conservation Area in northern Tanzania are at an advantage in developing small-scale farms to boost their economy by taking advantage of these deserted eggs.

## Conclusion

In this study, ostriches were found to be hunted in the wild for meat, eggs, feathers and oil, which have various uses, including economic and medicinal purposes. An increase in the utilization of ostrich products and the associated economic benefits suggests that conservation efforts are important and that there is a need to conserve and protect the apparently decreasing wild ostrich populations through the combined involvement of conservation authorities and the government. Wildlife management is an important complement to ostrich farming, and because the Tanzanian government allows game farming (MNRT 2000), district authorities can follow the procedures for establishing ostrich farms in their respective villages along with education centres focused on the farming of ostriches and the utilization of their products. Ostrich farming will only be effective if villagers are equipped with knowledge on farming and how to venture into the market to meet the demand. Ostrich farming will potentially reduce the pressure on the wild population but will also provide opportunities for livelihood improvement**.**
